# Are Phage Lytic Proteins the Secret Weapon To Kill *Staphylococcus aureus*?

**DOI:** 10.1128/mBio.01923-17

**Published:** 2018-01-23

**Authors:** Diana Gutiérrez, Lucía Fernández, Ana Rodríguez, Pilar García

**Affiliations:** a Instituto de Productos Lácteos de Asturias (IPLA-CSIC), Villaviciosa, Asturias, Spain; Harvard Medical School

**Keywords:** *Staphylococcus aureus*, bacteriophage, bacteriophage therapy, endolysin

## Abstract

Methicillin-resistant *Staphylococcus aureus* (MRSA) is one of the most threatening microorganisms for global human health. The current strategies to reduce the impact of *S. aureus* include a restrictive control of worldwide antibiotic use, prophylactic measures to hinder contamination, and the search for novel antimicrobials to treat human and animal infections caused by this bacterium. The last strategy is currently the focus of considerable research. In this regard, phage lytic proteins (endolysins and virion-associated peptidoglycan hydrolases [VAPGHs]) have been proposed as suitable candidates. Indeed, these proteins display narrow-spectrum antimicrobial activity and a virtual lack of bacterial-resistance development. Additionally, the therapeutic use of phage lytic proteins in *S. aureus* animal infection models is yielding promising results, showing good efficacy without apparent side effects. Nonetheless, human clinical trials are still in progress, and data are not available yet. This minireview also analyzes the main obstacles for introducing phage lytic proteins as human therapeutics against *S. aureus* infections. Besides the common technological problems derived from large-scale production of therapeutic proteins, a major setback is the lack of a proper legal framework regulating their use. In that sense, the relevant health authorities should urgently have a timely discussion about these new antimicrobials. On the other hand, the research community should provide data to dispel any doubts regarding their efficacy and safety. Overall, the appropriate scientific data and regulatory framework will encourage pharmaceutical companies to invest in these promising antimicrobials.

## INTRODUCTION

*Staphylococcus aureus* is one of the most important human pathogens, causing a variety of diseases (skin, soft tissue, wound, bone, and bloodstream infections, toxic shock syndrome, and food poisoning). This bacterium has become a serious threat in hospitals, as it is one of the most common causes of nosocomial infections. Moreover, the emergence of and increase in antibiotic resistance (especially methicillin resistance) in clinical environments are really worrying. Recent data from the World Health Organization (WHO) indicate that methicillin-resistant *S. aureus* (MRSA) strains are responsible for more than 20% of all infections in WHO regions, but this percentage can reach 80% in some countries (1).

Additionally, *S. aureus* is one of the major causative agents of food-borne diseases in humans due to the production of enterotoxins (2). In 2014, consumption of food products contaminated with *S. aureus* was responsible for 7.5% of all food-borne outbreaks in the European Union (EU) (3). The presence of MRSA in farm animals is also a serious concern, since animals can acquire and disseminate strains other than livestock-associated MRSA (4). It is well known that the widespread use of antibiotics in food animal production has favored the increase in multidrug-resistant bacteria (MDR) that led to the current global health crisis (5, 6). To cope with this problem, several countries have restricted the use of antimicrobials in animal farming (e.g., growth promoters and disease prevention compounds) (7).

**FIG 1:**
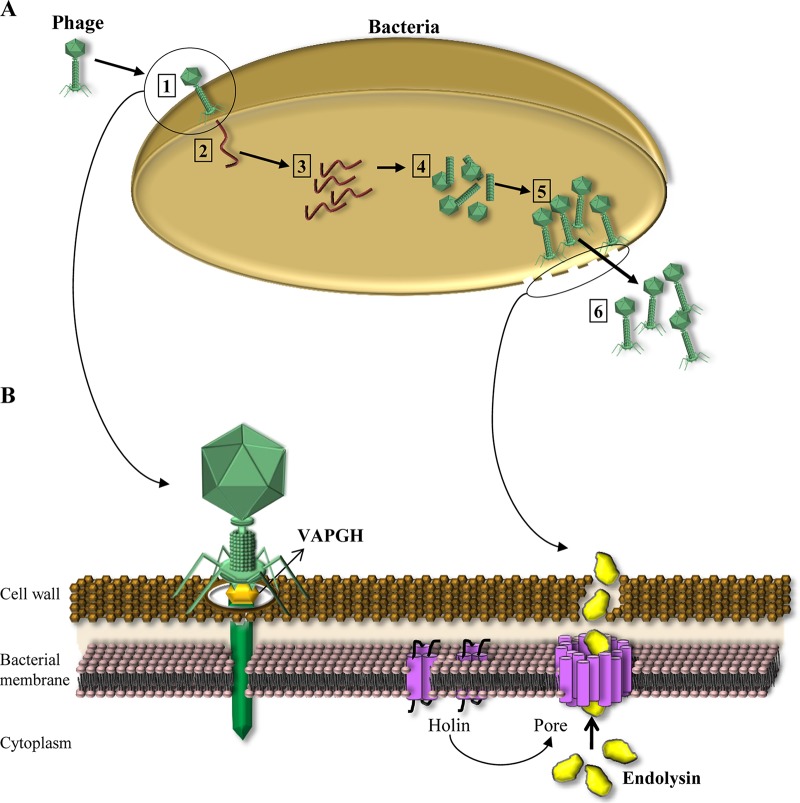
(A) Bacteriophage lytic cycle. 1, Adsorption of phage to the bacterium; 2, injection of genetic material into the cytoplasm; 3, replication of phage genetic material; 4, synthesis of phage components; 5, assembly of new phage particles; 6, bacterial lysis and release of phage particles. (B) Role of phage lytic proteins in the phage life cycle. VAPGHs favor the injection of phage genetic material into the cytoplasm by the formation of a hole in the cell wall. Endolysins and holins are produced at the end of the life cycle. Holins form a pore in the bacterial membrane, allowing the endolysin to reach the peptidoglycan.

Bacteriophages, or phages, are viruses that exclusively infect bacteria as they carry out their life cycle (Fig. 1A). In most cases, the lytic life cycle ends with the death of the bacterial cell, thereby making phages the natural killers of bacteria. Lysis can proceed by one of two basic mechanisms. On the one hand, phages with a single-stranded genome encode a lysis effector which inhibits the biosynthesis of bacterial peptidoglycan. In contrast, release of the phage progeny in double-stranded DNA (dsDNA) phages is mediated by two proteins, holin and endolysin, which are responsible for cell envelope disruption. Once the lytic life cycle has been completed and the virion particles are mature inside the bacterial cell, the holin forms pores in the inner cell membrane, allowing access of the endolysin to the cell wall. Subsequently, endolysin molecules degrade peptidoglycan, which is followed by osmotic lysis of the cell (Fig. 1A and B). In addition, several phages can use the host cell secretion machinery (Sec system) to release their endolysins and also encode a holin (pinholin) involved in proton motive force dissipation to activate the secreted endolysin. Phages infecting Gram-negative hosts are provided with additional proteins, named spanins, that help to break the outer membrane (8).

Virion-associated peptidoglycan hydrolases (VAPGHs) are structural components of the virion particle and participate in the initial steps of infection by slightly degrading peptidoglycan to allow entry of the phage genetic material into the bacterial cell (Fig. 1A and B). Both types of lytic proteins, endolysins and VAPGHs, are useful as antimicrobials due to their potential for degrading peptidoglycan, resulting in cell lysis when added exogenously. Recently, there has been a renewed interest in studying and exploiting the potential of phages and phage lytic proteins to combat undesirable bacteria (9–11). Additionally, phages can be used as tools for multiple health-related applications, including vaccine development, gene delivery, and bacterial detection (12).

In this context, this minireview aims to present and analyze the main advantages of phage lytic proteins to combat *S. aureus* in balance with the main obstacles that interfere with their commercialization.

## MAIN CHARACTERISTICS AND PROPERTIES OF *S. AUREUS* PHAGE LYTIC PROTEINS

### (i) Structure and enzymatic activity.

All phage lytic proteins (endolysins and VAPGHs) encoded by *S. aureus* bacteriophages have a modular structure, a common trait displayed by endolysins from Gram-positive dsDNA phages (13). This modular organization in distinct functional domains provides phage lytic proteins with two useful properties. On the one hand, this structure confers remarkable substrate specificity (further explained at the end of this section), and on the other hand, it allows the performance of protein engineering in order to design new proteins with enhanced antimicrobial activities (see the next section).

**FIG 2:**
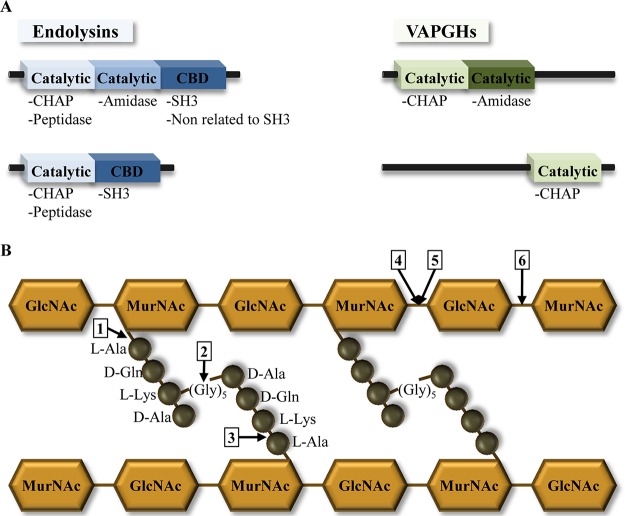
Structure and enzymatic activities of phage lytic proteins against *S. aureus* peptidoglycan. (A) The typical modular structure of phage lytic proteins (endolysins and VAPGHs) is represented by the catalytic domains and the cell wall binding domains (CBDs). (B) The structure of *S. aureus* peptidoglycan is shown, and the enzymatic activities of the proteins are indicated with an arrow and a number. 1, *N*-Acetylmuramoyl-l-alanine amidase; 2, interpeptide bridge endopeptidase; 3, l-alanoyl-d-glutamate endopeptidase; 4, *N*-acetyl-β-d-muramidase; 5, transglycosylase; 6, *N*-acetyl-β-d-glucosaminidase.

Most staphylococcal phage endolysins possess one or two N-terminal catalytic domains and one C-terminal cell wall binding domain (CBD). Interestingly, no signal peptides or transmembrane domains have been described in staphylococcal phage endolysins. A similar modular structure, consisting of one or two catalytic domains, was described for VAPGHs, although these proteins always lack a known CBD (11) (Fig. 2B).

In order to understand the catalytic activities of phage lytic proteins, it is important to look at the structure of their enzymatic target, bacterial peptidoglycan, which consists of linear glycan strands cross-linked by short peptides. These glycan strands are made up of alternating *N*-acetylglucosamine (GlcNAc) and *N*-acetylmuramic acid (MurNAc) residues linked by β-1,4 glycosidic bonds. The d-lactoyl group of each MurNAc residue is replaced with a peptide stem, whose composition in *S. aureus* is l-Ala-d-Glu-l-Lys-d-Ala. Cross-linking of the glycan strands generally occurs between the carboxyl group of d-Ala at position 4 and the amino group of the di-amino acid at position 3 through a short peptide bridge composed of five Gly residues (14) (Fig. 2B).

The catalytic domains of phage lytic proteins are classified into 6 different types according to their enzymatic activities against peptidoglycan: *N*-acetylmuramoyl-l-alanine amidases, interpeptide bridge endopeptidases, l-alanoyl-d-glutamate endopeptidases, *N*-acetyl-β-d-muramidases, transglycosylases, and *N*-acetyl-β-d-glucosaminidases (Fig. 2B). On the one hand, lysozymes (or muramidases) and transglycosylases cleave the *N*-acetylmuramoyl-β-1,4-*N*-acetyl-glucosamine bond, while glucosaminidases and amidases hydrolyze the *N*-acetylglucosaminyl β-1,4-*N*-acetylmuramine bond and the amide bond between the sugar and the peptide moieties, respectively. Finally, endopeptidases cleave the bond within the interpeptide bridge.

Endolysins from staphylococcal phages rarely contain transglycosylases. Instead, the catalytic domains found in these proteins are LYSO (phage lysozyme domain), PET-M23 (peptidase domain M23), AMI-2 (amidase 2 domain), AMI-3 (amidase 3 domain), and CHAP (cysteine- and histidine-dependent amidohydrolase/peptidase), with CHAP being the most frequent domain (>74%) (13).

Regarding CBDs, endolysins derived from phages infecting *S. aureus* usually contain SH3-related domains (accession number PF08460), with SH3_5 and SH3b being the most common (13, 15) (Fig. 2). SH3b domains have been shown to bind to the peptidoglycan peptide cross-bridge (16). However, there are some endolysins derived from phages phiNM3, phi13, and MW1 where the CBD showed no homology to SH3b (17). Daniel et al. (17) postulated that the phiNM3 CBD may bind to cell wall-associated carbohydrates instead of the pentaglycine peptide cross-bridge. More recently, a new type of CBD has been described in the endolysin of phage SA97 (LysSA97), which shares only 19% homology with other staphylococcal endolysins deposited in databases (18).

Most phage endolysins possess high specificity against the genus or species infected by the phage from which they derive, which represents a notable advantage over classical wide-spectrum antibiotics. Nonetheless, the interaction of these proteins with their substrate at the molecular level is not fully understood, so it is still not clear which fragment of the molecule determines specificity. There are limited data regarding the role of the catalytic domains in the specificities of endolysins. For example, fusion of catalytic domains from the endolysin encoded by *Streptococcus agalactiae* bacteriophage B30 to a CBD specific for *S. aureus* strains can expand the lytic activity of the chimeric protein to *S. aureus* (19). This suggests that catalytic domains do not exert strict specificity. Regarding CBDs, Becker et al. in 2009 showed that a chimeric protein consisting of the LysK SH3b domain and the streptococcal endolysin λSA2 catalytic domain exhibited both staphylolytic and streptolytic activities (15). Therefore, the specificities of endolysins might result from the combined interactions of catalytic and binding domains with species-specific cell wall receptors in the peptidoglycan structure, which remain unknown to date. Indeed, analysis of the crystal tridimensional structures of the individual domains might be useful for revealing these interactions and for designing site-directed mutants with altered activity or substrate specificity (20, 21).

### (ii) Design of chimeric proteins.

**FIG 3:**
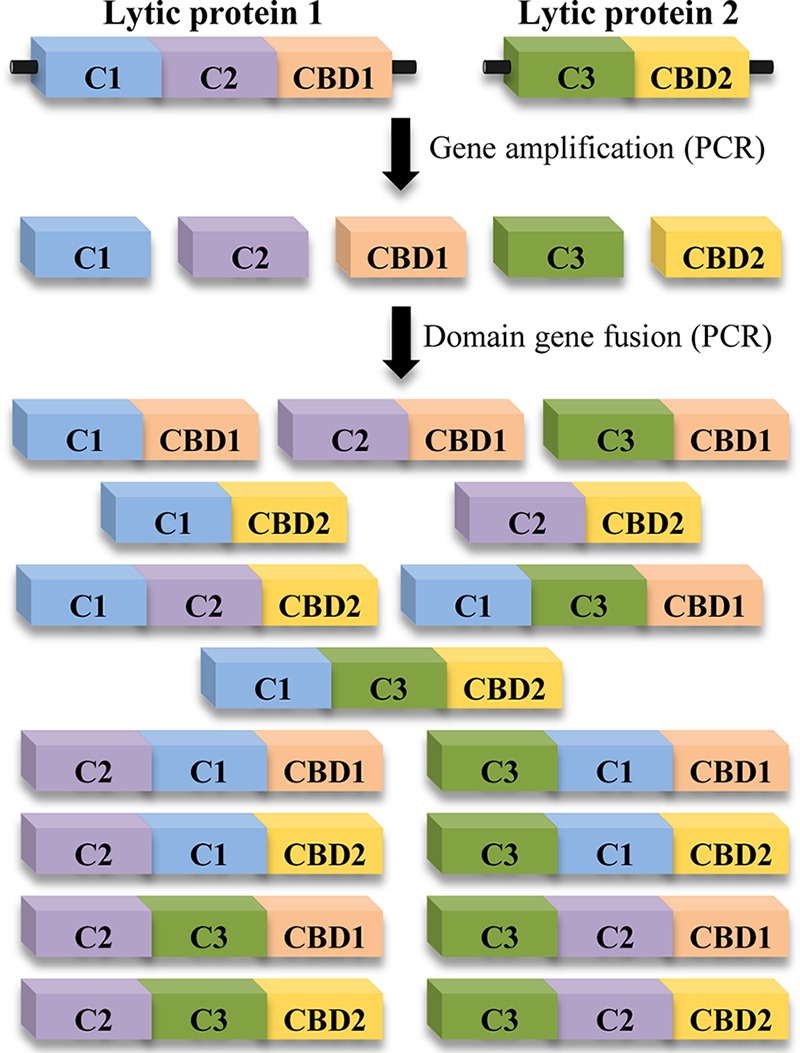
Schematic representation of the domain shuffling strategy to obtain chimeric proteins from two phage lytic proteins. C1, C2, and C3 represent catalytic domains, while CBD1 and CBD2 represent different cell wall binding domains.

The development of phage lytic proteins as novel antimicrobials entails systematic mining of naturally occurring proteins, as well as the design of new ones. This process is greatly facilitated by the modular structure of staphylococcal phage lytic proteins. Indeed, this organization allows exploring domain deletion and shuffling as a route to obtain new endolysins with enhanced properties (Fig. 3). Some of the strategies used in the design of new lytic proteins include the synthesis of truncated proteins, such as those containing only one catalytic domain or one CBD, and the production of new chimeric enzymes by combining domains from different lytic proteins. Thus, studies involving truncated proteins have demonstrated that enzymes containing just the CHAP domain of the parental endolysin generally display a slight increase in activity, whereas those containing only the amidase domain frequently have no lytic activity (22–24). Interestingly, the design of chimeric proteins has shown good results with regard to the development of improved lytic proteins. For instance, a chimeric protein based on LysK, PRF-119, was obtained by fusing the CHAP domain from LysK to the SH3b domain from lysostaphin (25). PRF-119 displayed very good activity (i.e., the MIC_90_ was 0.391 µg/ml for both MRSA and methicillin-susceptible *S. aureus* [MSSA] strains). Another example is a derivative of endolysin Ply187 containing the CHAP domain fused to the SH3b CBD of endolysin LysK, which exhibited a 10-fold increase in specific activity over that with the truncated protein carrying the individual CHAP domain (26). Similar results were obtained in studies that analyzed whether the presence of more catalytic domains in a single endolysin molecule leads to a higher activity. To test this hypothesis, chimeric proteins containing three catalytic domains plus one CBD were obtained by fusing the two LysK catalytic domains with the complete lysostaphin molecule. Unexpectedly, the resulting proteins showed intermediate activities compared with those of the respective parental proteins; i.e., for *S. aureus* USA100, the MIC values were 14 µg/ml and 20 µg/ml for the chimeric proteins K-L and L-K, respectively, which are between those determined for lysostaphin (1.2 µg/ml) and LysK (96 µg/ml) (2,7).

Finally, an important step forward in the study of chimeric proteins against *S. aureus* was the modification of phage lytic proteins to kill intracellular *S. aureus*. This was achieved by using a protein transduction domain (PTD) composed of a short cationic peptide sequence that facilitates crossing of the eukaryotic membrane by the lytic protein. The same study also showed that lysostaphin requires the presence of a PTD for the eradication of intracellular *S. aureus*. However, some chimeric proteins derived from LysK and lysostaphin did not require this domain to enter cultured bovine mammary cells (27).

From all these data, we can conclude that domain shuffling is a powerful tool for increasing the activities of phage lytic proteins. Of note, it has been widely proven that CHAP domains from *S. aureus* phage lytic proteins possess higher activity than other types of catalytic domains, at least under *in vitro* conditions (22–24). Moreover, the addition of a CBD to an individual CHAP domain significantly increases its *in vitro* activity (91).

### (iii) Antibiofilm activity.

One of the main obstacles for the eradication of *S. aureus* in hospitals and food industries is its ability to form biofilms. These structures are the most common lifestyle of bacteria in nature. *S. aureus* is not an exception, and most strains show varied abilities to adhere to and grow on several biotic and abiotic surfaces (28). Worryingly, biofilms represent a barrier to the actions of antibiotics and disinfectants, hindering both the treatment of infections and the cleaning of surfaces. In this context, there is considerable evidence with regard to the efficacy of phage lytic proteins against preformed *S. aureus* biofilms. Some of the proteins with proven antibiofilm properties are endolysins SAL-2, phi11, PlyGRCS, and SAL200, as well as the chimeric proteins Chapter K (derived from LysK), ClyH (derived from the Ply187 and phiNM3 endolysins), and ClyF (derived from the Ply187 and PlySs2 endolysins) (29–35). In the case of the chimeric protein ClyH, the susceptibilities of biofilms turned out to be dependent on the strain and the biofilm maturation stage. Thus, removal of 72-h-old biofilms needed a longer treatment than removal of 24-h- and 48-h-old biofilms, probably due to the presence of a greater amount of extracellular material (29). However, complete removal of adhered cells in biofilms is not easy to achieve by using phage lytic proteins. To solve this problem, some authors have proposed the use of two consecutive rounds of treatment (36) and/or combination with antibiotics. For instance, treatment of *S. aureus* biofilms with minocycline followed by treatment with endolysin MR-10 can significantly reduce both young and mature biofilms formed by MRSA (37). The main advantage of endolysins as antibiofilm agents over traditional antibiotics is their ability to lyse bacteria even when they are not actively growing. In addition, endolysin LysH5 was proven to be active against persister cells, which also contribute to the recalcitrant nature of biofilms (36).

The development of products based on phage proteins to eliminate bacterial biofilms requires accurate quantification of the antibiofilm activities exhibited by different proteins. Indeed, such a technique is of paramount importance in selecting those proteins that display the highest activity. Recently, a method that measures biofilm formation and development in real time was validated to establish the antibiofilm activities of phage lytic proteins. This method relies on changes in the impedance signals caused by *S. aureus* when attaching and detaching after protein treatment (38).

Besides in biofilm eradication, phage lytic proteins can be useful for the inhibition of biofilm development. For instance, a feasible strategy that can be applied in the near future to prevent the attachment of *S. aureus* to surfaces is manufacturing antimicrobial surfaces coated with endolysins. In fact, lytic proteins can be attached to silica nanoparticles (SNPs) to facilitate surface incorporation or embedded into films of polyhydroxyethyl methacrylate, which has already shown efficacy against *Listeria* (39).

### (iv) Bacterial resistance and adaptive responses.

In addition to exhibiting high antibacterial activity, a good antimicrobial agent should preferably not select for bacterial resistance. To date, data about the emergence of resistance to endolysins in bacteria belonging to different genera indicate that resistance acquisition is quite rare or even nonexistent. Undoubtedly, this is one of the most valuable characteristic of endolysins and might be linked to the fact that their targets in the peptidoglycan molecule are essential for bacterial viability and fitness. As a result, mutations leading to endolysin resistance would be too harmful for the bacterial cell (40). Moreover, most *S. aureus* endolysins contain two catalytic domains, which theoretically would reduce the probability of finding bacteria with a double modification in the target structures. There have been several attempts to study the acquisition of resistance to phage lytic proteins in *S. aureus*, although no resistant bacteria were detected (41, 42). More recently, it was described that sublethal exposure of *S. aureus* to LysK in liquid medium yielded cultures for which the MIC increased 42-fold, while exposure in solid medium resulted in only a 2-fold increase in resistance (27). In contrast, *S. aureus* cells exposed to chimeric proteins formed by three catalytic domains (two catalytic domains from LysK fused to lysostaphin) showed hardly any increase in resistance. Indeed, the chimeric proteins K-L and L-K yielded cultures for which the MICs increased 8-fold and 2-fold, respectively, after exposure in liquid medium, whereas exposure in solid medium did not lead to a detectable increase in resistance (27). These observations support the importance of designing new chimeric proteins to improve the properties of natural endolysins and VAPGHs. In general, bacterial resistance development against phage lytic proteins is lower than that obtained for traditional antibiotics, although the frequency under *in vivo* conditions has not yet been determined. Besides resistance development, a recent study has evaluated the transcriptional response of *S. aureus* cells exposed to subinhibitory concentrations of phage lytic proteins. This study revealed that endolysin LysH5 and the VAPGH-derived chimeric protein CHAPSH3b led to the downregulation of genes encoding different proteins with autolytic activities (43). Fernández et al. linked these transcriptional changes to a decrease in biofilm formation, as the major autolysin AtlA is an important factor in early stages of biofilm development (43). This reinforces the usefulness of lytic proteins as antibiofilm compounds. Interestingly, this article also showed that deletion of the autolysin-encoding gene leads to low-level resistance to the two lytic proteins. This suggests that the gene expression changes triggered by lytic proteins may confer some degree of adaptive resistance to these antimicrobials, and therefore, this deserves to be evaluated before the extensive use of these proteins.

## THERAPEUTIC EFFICACY OF PHAGE LYTIC PROTEINS

### (i) Animal models of infection.

**FIG 4:**
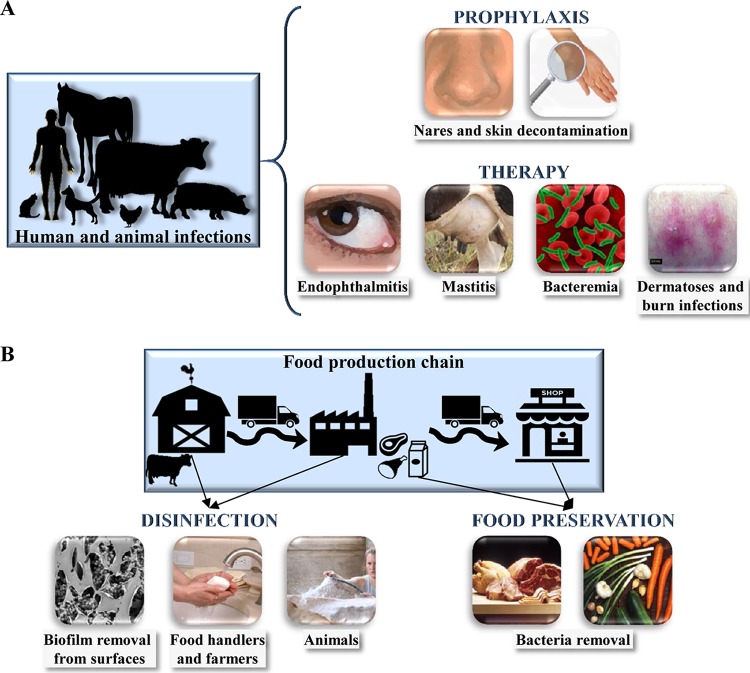
Current applications of *S. aureus* phage lytic proteins in human and animal therapy (A) and improvement of food safety (B).

After confirming the effectiveness of phage lytic proteins under *in vitro* conditions, it is essential to prove that they are also active *in vivo*. For this purpose, different animal models have been set up to mimic infections caused by *S. aureus*. These models allow testing the efficacy of therapeutic and prophylactic treatments of these infections with lytic proteins (Table 1; Fig. 4A). Prophylaxis is particularly relevant with regard to *S. aureus* due to its presence on human skin, which constitutes a danger for patients with chronic diseases, immunocompromised patients, and also for those subjected to surgery or hemodialysis. In some countries, nasal decolonization in high-risk patients is currently carried out using mupirocin. However, effective removal of *S. aureus* from the nose requires the administration of several subsequent doses over 5 days, which raises the concern of mupirocin resistance development (44). In this context, several phage lytic proteins have been assayed in mice and rats to remove *S. aureus* from previously contaminated nostrils (27, 41, 45–47) (Table 1). Another potential prophylactic application of endolysins is skin decolonization of clinical patients, healthy workers, or food handlers. The effectiveness of this measure has been evaluated by using skin models (porcine and murine) colonized by *S. aureus*, where lytic proteins were applied by spraying or as an emollient ointment (33, 41) (Table 1).

**TABLE 1:** Phage lytic proteins against *S. aureus* and their most relevant properties

Application(s)	Lytic protein(s)	Origin	Result(s)	Reference(s)
Prophylaxis	P128	CHAP domain (TAME phage K) + SH3b (lysostaphin)	In rat nares, there was a 4-log reduction after treatment with 15 µg of protein	45
Prophylaxis	L-K	Catalytic domain (LysK) + lysostaphin	In mouse nares, there was a 98% reduction after treatment with 200 µg of protein	27
Prophylaxis with Chapter K	CHAP domain (LysK)		In mouse nares, there was a 2-log reduction after 1 h of treatment with 925 μg of protein; in porcine skin (colonized by 2.5 × 10^5^ CFU/cm^2^), there was a 99% reduction after 30 min of treatment	33, 46
Prophylaxis, bacteremia	ClyS	Catalytic domain (phage Twort) + CBD (phiNM3)	In mouse nares, there was a 2-log reduction after treatment with 960 µg of protein; in mouse skin, there was a 3-log reduction; intraperitoneal injection (1 mg) at 3 h postinfection resulted in 88% survival; combination with vancomycin and oxacillin resulted in 100% survival	17, 41
Prophylaxis, bacteremia	MV-L	Phage MV-L	In mouse nares, there was a 2 × 10^9^ CFU reduction after treatment with 310 U of protein; intraperitoneal injection (500 U) for 60 min after infection resulted in 60% survival	47
Bacteremia	SAL-1 (SAL200)	Phage SAP-1	In a mouse model, intravenous administration (25 mg/kg) resulted in 100% survival	32
Bacteremia	LysGH15	Phage GH15	Intraperitoneal injection (50 µg) 1 h after infection resulted in 100% survival	48
Bacteremia	Phi11, 80a, LysK, 2638A, WMY, Twort, phiSH2	Phage phi11, phage phi80a, phage K, phage 2638A, phage WMY	In a mouse model, intraperitoneal injection (200 µg) 30 min postinfection resulted in 100% survival for phi11, 80a, LysK, 2638A, and WMY and a 50 to 60% reduction for Twort and phiSH2	50
Bacteremia	P-27/HP	Endolysin from phage P-27/HP	In a mouse model, intraperitoneal injection (250 µg) after 24 h of infection resulted in a 99.9% reduction in CFU counts in spleens	51
Bacteremia	CF-301		With the endolysin *Streptococcus suis* prophage, the combination of CF-301 (1.25 mg/kg) and daptomycin 4 h after infection yielded 73% survival	55
Bacteremia, burn infection	ClyF	CBD domain (PlySs2) + catalytic domain (Ply187)	Intraperitoneal injection (50 mg/kg) at 3 h postinfection resulted in 100% survival after bacterial reduction of 1.5 to 3.3 log_10_ in the treatment of burn infections with 0.1 mg at 24 h postinfection	35
Disinfection, bacteremia	MR-10	Phage MR-10	Biofilm reduction after sequential treatment with minocycline (4 μg/ml) for 3 h followed by treatment with endolysin MR-10 (18 to 36 μg/ml) for 16 h; the combination of MR-10 and minocycline resulted in a 100% survival	37, 49
Endophthalmitis	Ply187	CHAP domain (Ply187) + SH3b (LysK)	In a mouse model, intravitreal injection resulted in a 1- to 2-log reduction at 6 h and 12 h after infection	54
Mastitis	Trx-Sa1	Phage IME-SA1	In cows, bacterial counts were reduced to undetectable levels after 3 days	53
Mastitis	λSA2-E-Lyso-SH3b, λSA2-E-LysK-SH3b	Endopeptidase domain (streptococcal SA2 endolysin) + CBD (lysostaphin or LysK)	In a mouse model, there were 0.63- and 0.81-log-unit reductions after gland infusion (25 µg) and a synergistic effect with lysostaphin	52
Dermatoses	Staphefekt, SA.100	Commercial endolysin	In humans, there was a reduction of inflammatory symptoms of osteosarcoma, impetigo, and folliculitis after a 2-week application twice a day	60
Food preservation	LysSA97	Phage SA97	In beef and milk, there was a 0.8 ± 0.2-log reduction in the no. of CFU/ml; there was also a reduction of 4.5 ± 0.2-log CFU/ml when LysSA97 was combined with carvacrol	62
Food preservation	HydH5, HydH5Lyso, HydH5SH3b, CHAPSH3b	VAPGH-derived proteins (phage phiIPLA88)	In milk, bacteria were undetectable (10^4^ CFU/ml) after 6 h of incubation at 37°C	64
Disinfection, food preservation	LysH5	Phage phiIPLA88	Biofilm was removed after treatment with 0.15 µM for 6 h, followed by a second treatment for 12 h; in pasteurized milk, bacteria were undetectable (10^6^ CFU/ml) after treatment with 88 μg/ml for 4 h; there was a synergistic effect with nisin	36, 63, 65

Regarding infection treatment with phage lytic proteins, bacteremia has been the most widely studied, probably because it is the most dangerous stage in the *S. aureus* infection process. For example, induction of bacteremia in a mouse model by intraperitoneal injection of *S. aureus* (10^9^ CFU/mouse) resulted in a mortality rate of 100% within 3 days. However, administration of a single intraperitoneal or intravenous injection of a solution containing a phage lytic protein significantly improved the survival of mice (17, 32, 35, 37, 47–51) (Table 1). Moreover, this treatment significantly reduced the inflammatory response caused by bacteremia (48). Thus, animals treated with the lytic proteins exhibited normal levels of the cytokines gamma interferon (IFN-γ), interleukin 4 (IL-4), and IL-6 mRNA. However, it must be noted that different lytic proteins displayed various degrees of antimicrobial activity. For example, a study described that six lytic proteins (80a, phi11, LysK, lysostaphin, 2638A, and WMY) provided total protection from bacteremia-induced death but that Twort or phiSH2 conferred only partial protection (50).

Phage lytic proteins can also be used for the treatment of mastitis in farm animals. To determine the feasibility of this application, a mouse model of mastitis was developed by infusion of 10^2^ and 10^4^ CFU into the mammary glands and subsequent treatment with several lytic proteins (52). This treatment led to a significant reduction in bacterial cell counts (Table 1). To date, the results available regarding therapeutic trials with endolysins in cow udders are only preliminary but promising (53) (Table 1). Clearly, additional studies are needed before commercialization of these antimicrobials for application in cattle. Nonetheless, phage lytic proteins bring a positive outlook about the future of infection control in animal farming without contributing to the rise in antibiotic resistance.

Finally, the application of endolysins for the treatment of ocular infections after surgery has also been explored, with good results (54) (Table 1). All these data confirm the *in vivo* efficacy of phage lytic proteins against *S. aureus* infections and suggest that lytic proteins do not trigger a significant immune response. Nevertheless, it is important to highlight that lytic activities vary greatly between proteins, leading, in some cases, to the need to administer a high-protein concentration. This may have consequences for the immune response and needs to be carefully examined on a case-by-case basis.

### (ii) Combination therapy with phage lytic proteins and other antimicrobials.

A strategy to improve the activities of phage lytic proteins is to combine them with other antimicrobials, which may lead to a synergistic effect against the target bacteria (17, 49, 55) (Table 1). This is the case of the lytic protein CF-301 and daptomycin, whose combination significantly increased survival from bacteremia in mice compared to levels of survival with the two antimicrobials used separately (55). Similarly, a combination of endolysin MR-10 and minocycline reverted systemic MRSA infection in mice, resulting in 100% survival, and improved treatment of localized burn wound infections (49). Moreover, phage lytic proteins also displayed increased antimicrobial activity when combined with other proteins that hydrolyze peptidoglycan bonds. In a mouse model of mastitis, for instance, the chimeric protein λSA2-E-LysK-SH3b showed a synergistic effect with lysostaphin against *S. aureus* (52) (Table 1). A clear advantage of combination therapy is that the synergistic effect allows reducing the doses of each antimicrobial, thus limiting possible side effects. Moreover, this strategy may also theoretically reduce the likelihood of development of bacterial resistance to antimicrobials with different mechanisms of action.

### (iii) Safety studies.

Before a new drug can be used for therapeutic purposes in humans, several preclinical and clinical trials have to be performed. Preclinical studies include pharmacokinetics, ADME (absorption, distribution, metabolism, and elimination), and other safety-related parameters, such as genotoxicity, mutagenicity, safety pharmacology, and general toxicology (56). Among all these requirements, only a few have already been met for *S. aureus* phage lytic proteins. For instance, the cytotoxicity of phage lytic protein P128 against two cell lines, HEp2 and Vero, was evaluated by using the 3-(4,5-dimethyl-2-thiazolyl)-2,5-diphenyl-2H-tetrazolium bromide (MTT) colorimetric assay to test any reduction in viability. No cytotoxic effect was observed even at the highest concentration tested (2.5 mg/ml), which corresponds to more than 100× the MIC of this protein against *S. aureus* (57).

Additionally, the safety of phage lytic proteins was evaluated by studying whether they induced an inflammatory response and/or toxicity in animal models. A study showed that repeated treatment of mice with MV-L protein (500 U) via intraperitoneal injection triggered an immune response displayed as an increase in the level of antibodies against this protein (47). However, there were no apparent adverse effects for the animals or reduction in the antimicrobial activity of the protein (47). Moreover, repeated topical application of the lytic protein ClyS resulted in a low production of antibodies, and there was no inhibition of the lytic activity of the protein (17).

The toxicities of these proteins, as well as their intraperitoneal injection and topical application, have been studied after single- and repeated-dose intravenous administration. Phage lytic protein SAL200 was intravenously administered (2 to 100 mg/kg of body weight) in mice and dogs, in general, with no abnormal findings. In safety pharmacology studies, some abnormalities were observed in dogs after several doses (usually when protein injection was performed for more than 1 week after the initial administration), which disappeared without damage to the cardiovascular, respiratory, and central nervous systems (58).

Another interesting study carried out pharmacokinetic and safety tests on lytic proteins in monkeys. The maximum protein concentration in serum occurred immediately after administration and ranged from 40.5 to 378.4 μg/ml, the mean residence time being approximately 1 h. Another parameter that should be taken into consideration is the terminal half-life, defined as the time that it takes for a substance to lose half of its pharmacological activity. The values determined so far range from 0.4 to 5.3 h in males and 0.3 to 3.4 h in females after 1 day, whereas the terminal half-life ranged from 1.8 to 9.7 h in males and 1.2 to 5.3 h in females after 5 days. This study also assessed the safety and tolerability of SAL200 endolysin after intravenous administration of a single dose of 1 to 80 mg/kg/day for 6 days or multiple doses of 40 mg/kg/day for 5 days. The protein was well tolerated, and no adverse effects were detected (59).

Keeping all these results in mind, it seems that in order to avoid any harmful effects, the dosing period for phage lytic proteins should be shorter than 1 week and the dose should be as low as possible within the efficacy range of the protein. In any case, further studies are still needed to totally ensure the safety of these proteins.

### (iv) Human clinical trials and product pipeline.

The use of phage lytic proteins for the treatment of human infections is by far the research field of greatest interest among all possible applications of these antimicrobials. Nonetheless, only one endolysin-based product is currently on the market. The Dutch biotech company Micreos has developed the first product containing an endolysin for human use, Staphefekt, which is specific against *S. aureus*, including MRSA strains. This product is recommended for the early stages of *S. aureus*-related skin infections, such as eczema, acne, and rosacea, resulting in a reduction of inflammatory symptoms. Several formulations, such as creams and gels, are currently commercialized under the Gladskin brand. It has recently been demonstrated that this product can successfully treat chronic and recurrent *S. aureus*-related dermatoses without generating bacterial resistance after long-term daily therapy (60).

Protein SAL200 was recently assessed in the first in-human phase 1 study of a phage endolysin-based drug to be administered intravenously. This work consisted of a single-center, randomized, double-blind, placebo-controlled, single-dosing, and dose-escalating study of intravenous SAL200 administration in healthy male volunteers. No serious adverse effects were observed (61).

The company ContraFect recently started performing studies with CF-301, which is thus far the only endolysin to have entered human clinical trials in the United States. This protein has been specifically formulated for the treatment of *S. aureus* bloodstream infections, including endocarditis. Phase 1 clinical trials performed with healthy volunteers showed no adverse effects when the protein was administered intravenously. The company initiated phase 2 clinical trials with bacteremia patients in mid-2017 (https://clinicaltrials.gov/ct2/show/NCT03163446#wrapper).

The Indian company GangaGen is working with the lytic protein P128 (StaphTAME), which is intended for clearing nasal contamination of *S. aureus* in humans and is currently undergoing phase 2 clinical trials. The goal of these clinical trials is to determine the protein’s pharmacokinetics, immunogenicity, safety, and tolerability in healthy adult volunteers. In a second study focused on assessing the safety and efficacy of this product, the protein was applied to the anterior nares of *S. aureus* carrier patients with chronic kidney disease who were on dialysis and also to clinical patients in stable condition (https://www.clinicaltrials.gov/ct2/show/NCT01746654?term=NCT01746654&rank=1). The company also plans to investigate the treatment of infected venous ulcers, since a preliminary study has already shown the safety of the protein for topical application.

Apart from the above-mentioned products, there is an important number of companies working on the development of phage-based products for medical applications (http://companies.phage.org/), some of which are already in different phases of clinical trials. Satisfactory results in these clinical trials might pave the way for research concerning new phage-derived proteins.

## *S. AUREUS* PHAGE LYTIC PROTEINS FOR THE IMPROVEMENT OF FOOD SAFETY

The eradication of *S. aureus* from food environments (farm animals, industry surfaces, food handlers) (Fig. 4B) is indispensable to avoid contamination during processing of foodstuffs. Phage lytic proteins offer an alternative to classical biopreservation techniques (62–65) (Table 1) and contribute to the improvement of cleaning and disinfection procedures [see “(iii) Antibiofilm activity” above]. For instance, endolysin LysH5 was able to reduce *S. aureus* contamination in pasteurized milk down to undetectable levels (65). Moreover, it was shown that the ability of these proteins to remove pathogenic bacteria can be improved by combination with other food additives, such as essential oils and bacteriocins. For instance, the combination of endolysin LysSA97 and carvacrol reduced *S. aureus* contamination below the detection limit in both pasteurized skim milk and beef (62). Similarly, a synergistic effect between LysH5 and the bacteriocin nisin was observed for the elimination of *S. aureus* in milk (63). Synergy between phage lytic proteins and other antimicrobials might be explained by an initial weakening of the cell wall caused by the endolysin, which might facilitate the subsequent entry of the antibiotic or bacteriocin inside the bacterial cell (56).

One important issue regarding the use of phage lytic proteins as biopreservatives is the effect on the protein effectiveness of the physicochemical composition of the food matrix (66) and the strategy followed to deliver the protein into the food. One example of the latter is the use of starter cultures secreting endolysin to avoid contamination during the cheese manufacturing process. For example, *Lactobacillus casei* BL23 was engineered to deliver endolysin Lysdb to keep low levels of *S. aureus* contamination during production of cheese made from raw milk (67).

Finally, the ability to detect very low concentrations of pathogenic bacteria in food is very relevant. In this regard, the CBDs from endolysins offer a good opportunity to create specific biosensors with high affinity and specificity. For example, the CBD from endolysin plyV12 was used to obtain coated beads able to concentrate *S. aureus* cells by an inmunomagnetic separation method, the detection limit in milk being 4 × 10^3^ CFU/ml in a testing time of 1.5 h (68).

## CHALLENGES RELATED TO THE COMMERCIALIZATION OF PHAGE LYTIC PROTEINS

### (i) Large-scale production and formulation.

The widespread use of phage lytic proteins as antimicrobials would require large-scale production and proper purification of these proteins. In this context, the two main difficulties that must be overcome for translating from lab-scale to large-scale production are total production costs and safety issues. *Escherichia coli* is the most common bacterium used for the expression of recombinant proteins, since engineered strains are well known and there are several molecular tools set up to work with this bacterium (69). Alternatively, high recombinant protein yields can be obtained using the methylotrophic yeast *Pichia pastoris* or some filamentous fungi (*Aspergillus niger*, *Aspergillus oryzae*, *Aspergillus awamori*, *Chrysosporium lucknowense*, and *Acremonium chrysogenum*) (70).

To date, *S. aureus* endolysins have been purified mostly from *E. coli*, although it would be possible to assess other systems for their expression; an example is chloroplasts, which have the advantages that they lack endotoxins and have a low cultivation cost. Indeed, the endolysins cpl-1 and Pal, specific to *Streptococcus pneumoniae*, were successfully expressed in chloroplasts of *Chlamydomonas reinhardtii* (71). Moreover, a platform for expressing an endolysin against *Propionibacterium acnes* in cyanobacteria has recently been submitted for a patent (European patent application WO 2016130024). The main advantages of microalgae are low cost, easy upscaling, and the generally recognized as safe (GRAS) status of several species. Similarly, expression of recombinant proteins in plants is also a feasible alternative (72).

An important issue regarding proteins intended for therapeutic applications is the purification degree. Proteins must be highly purified, especially those to be administered parenterally. Suspensions of these proteins have to be sterile products obtained after a high number of phases, including centrifugation, ultrafiltration, and chromatographic steps, followed by sterilization by filtration and ultrafiltration. All these processes must be performed in clean areas (73).

Finally, the formulation of therapeutic compounds containing lytic proteins requires overcoming some issues, such as the stability of the proteins under storage conditions, compatibility with the route of administration, and reduction of immunogenicity. Thus, several formulations include the presence of polymers to avoid protein aggregation. For instance, endolysin SAL-1 was formulated using calcium ions and poloxamer 188 to prevent aggregation and to maintain stability during long-term storage (32). Similarly, the stability of LysK was improved by forming complexes with polycationic polymers, such as poly-l-lysines. The interactions between the protein and the polymers can break down enzyme aggregates, increasing the lytic activity and keeping full activity for at least 4 months (74). Similar results were obtained for the chimeric protein K-L (containing the LysK CHAP endopeptidase and amidase domains, as well as the lysostaphin glycyl-glycine endopeptidase domain), which showed increased stability in the presence of block copolymers of poly-l-glutamic acid and polyethylene glycol (75). On the other hand, incorporation of the lytic proteins into these polymers has the advantage of reducing their immunogenicity by hindering recognition by the immune system. This also prevents the inactivation of the protein caused in some cases by the polyethylene glycol used to reduce protein immunogenicity (75).

Regarding the use of lytic proteins for topical applications, e.g., skin decolonization or disinfection, products can be formulated as an ointment using the commercially prepared Aquaphor. This formulation contains 41% petrolatum and other ingredients, such as mineral oil, ceresin, lanolin alcohol, panthenol, glycerin, and bisabolol, and facilitates penetration of the phage lytic proteins into the skin (17).

Finally, the administration of phage lytic proteins might require a system to control their delivery, such as nanoencapsulation. Release of these lytic proteins can then be triggered by pH, temperature, redox gradients, ultrasound intensity, light, or electric pulses (76). Some successful results have been obtained with a cocktail containing the proteins Chapter K and lysostaphin, which were encapsulated in nanoparticles of poly-*N*-isopropylacrylamide (PNIPAM). The encapsulated proteins were released from the nanoparticles after a temperature increase, which simulated the conditions occurring in the skin during an *S. aureus* infection (77). Overall, it seems that both production and formulation issues regarding phage lytic proteins can easily be overcome in the near future, as has been shown previously for other types of therapeutic proteins.

### (ii) Regulatory framework.

To date, no phage lytic proteins have been accepted for human therapeutic use in Europe or the United States. The only exception is Staphefekt, which is the first endolysin-based product approved in the EU under the status of “medical device” (Medical Devices Directive 93/42/EC) (78). Aside from that, phage lytic proteins might be approved as “biological therapeutic proteins,” since they exhibit properties similar to those of other recombinant proteins that are already commercialized (79). Fortunately, this seems to be a shorter path than the one necessary for the authorization of bacteriophages, since there is no current legal framework that allows companies to place on the market bacteriophage products intended for human therapy (80).

In the United States, Food and Drug Administration (FDA) approvals for recombinant proteins have consistently increased since the 1980s. In general, biologics-based medicines have shorter authorization times than small molecules. For example, authorization of recombinant enzymes takes about 5.9 years versus the 8.3 years required for small molecules like antibiotics (81).

Another recent trend is the approval of some biologics under the Orphan Drug Act, which was initially set up to encourage the development of drugs for rare diseases. Orphan drugs have some advantages for their commercialization, as they have government financial incentives and smaller clinical trial sizes, shorter clinical trial times, and higher rates of regulatory success than standard drugs (82).

It is important to note that the development of a new drug is estimated to cost 2.6 billion U.S. dollars and to take at least a decade (83). This high cost, along with the short time of use of antibacterial agents by consumers, has made the development of these compounds less attractive to pharmaceutical industries.

In the EU, the requirements for marketing medical products for human and veterinary use are regulated by directive 2001/83/EC (84) and EC regulation 726/2004 (85). Within this framework, phage lytic proteins might be used in veterinary medicine for the treatment of *S. aureus* infections, such as mastitis in cows. The European Medicines Agency provides information for companies and individuals involved in developing and marketing medicines for veterinary use in the EU. Meanwhile, medical products without a marketing authorization can be used in patients with chronic diseases or when there is no other product available to treat the disease by means of the Compassionate Use Programs or Expanded Access Programs. Similarly, in the United States, House Resolution 878 (the Right to Try Act of 2017 [https://www.congress.gov/bill/115th-congress/house-bill/878/actions]), which allows patients who have been diagnosed with a terminal illness to be treated with drugs that have successfully completed phase 1 of clinical investigation, has been proposed.

When used as biopreservatives in the food industry, endolysins are considered food additives and regulated as such. In this regard, EC regulation 1333/2008 (86) states that all additives in the EU must be authorized and listed with their respective conditions of use in the EU’s positive list based on a safety assessment, technological need, and assurance that use of the additive will not mislead consumers.

Last, but not least, phage lytic proteins can also be used as disinfectants. In the EU, this requires approval under the Biocidal Products Regulation (BPR) (87). The regulations concerning disinfectant approval in the United States are the Federal Insecticide, Fungicide, and Rodenticide Act (FIFRA) (88) and the Medical Devices Amendments of 1976 (89) to the Federal Food, Drug, and Cosmetic Act (90). These two regulations are overseen by the Environment Protection Agency (EPA) and the FDA, respectively. FIFRA controls the commercialization of products for the disinfection of household and clinical contact surfaces, whereas the Medical Devices Amendment regulates the use of liquid chemical sterilants and high-level disinfectants used for disinfection of clinical devices. The existing regulatory frameworks in both the EU and the United States ensure that products are efficacious but exhibit low toxicity and environmental risks. As a result, application for this type of approval generally requires a large number of studies, leading to high costs and long processing times. In this scenario, the commitment by the authorities to facilitate the process of approval of these products, as well as the commitment of the companies to invest in product development, will be essential to place these new antimicrobials on the market.

## CONCLUDING REMARKS

Today, infectious diseases remain an important cause of death, a situation that can only worsen with the increase in the antibiotic resistance of pathogenic bacteria. Hopefully, the lessons learned since the time of Fleming will allow us to search for new antimicrobials with improved characteristics against refractory bacteria. A set of phage lytic proteins active against *S. aureus* are being studied for their application in urgent medical scenarios, such as bacteremia and endocarditis. The structure of these proteins offers many possibilities for their manipulation, and data about protein-substrate interaction will be very valuable in understanding their mechanisms of activity and specificity. An important issue that deserves further research is the potential development of bacterial resistance to phage lytic proteins. Although this does not seem particularly worrying at present, resistance selection under *in vivo* conditions still has to be studied. More efforts are also needed to find the most adequate combinations of phage lytic proteins and other antimicrobials able to totally remove *S. aureus* biofilms from both clinical and food environments. The adaptive responses of *S. aureus* biofilms exposed to phage proteins and their consequences regarding virulence and resistance should also be elucidated. Overall, it can be concluded that there is evidence of the effectiveness of phage lytic proteins as therapeutics in animal models of disease and as food biopreservatives, although efficacy in the latter application depends on the physicochemical properties of the food. On the basis of these successful results, preclinical studies and clinical trials are under way. However, it is clear that further data about drug safety are still necessary. Nonetheless, the support of pharmaceutical companies through investment in these antimicrobials is essential to definitely boost this research. In turn, this cannot be achieved without an incentive from health authorities, together with a new legal framework for authorization of these products.
